# Container Migration in the Fog: A Performance Evaluation †

**DOI:** 10.3390/s19071488

**Published:** 2019-03-27

**Authors:** Carlo Puliafito, Carlo Vallati, Enzo Mingozzi, Giovanni Merlino, Francesco Longo, Antonio Puliafito

**Affiliations:** 1 DINFO, University of Florence, Via di S. Marta 3, 50139 Florence, Italy; 2 Department of Information Engineering, University of Pisa, Largo Lucio Lazzarino 1, 56122 Pisa, Italy; carlo.vallati@iet.unipi.it (C.V.); enzo.mingozzi@unipi.it (E.M.); 3 Department of Engineering, University of Messina, Contrada di Dio, Sant’Agata, 98166 Messina, Italy; gmerlino@unime.it (G.M.); flongo@unime.it (F.L.); apuliafito@unime.it (A.P.)

**Keywords:** fog computing, internet of things, mobility, container, migration, CRIU, pre-copy, post-copy

## Abstract

The internet of things (IoT) is essential for the implementation of applications and services that require the ability to sense the surrounding environment through sensors and modify it through actuators. However, IoT devices usually have limited computing capabilities and hence are not always sufficient to directly host resource-intensive services. Fog computing, which extends and complements the cloud, can support the IoT with computing resources and services that are deployed close to where data are sensed and actions need to be performed. Virtualisation is an essential feature in the cloud as in the fog, and containers have been recently getting much popularity to encapsulate fog services. Besides, container migration among fog nodes may enable several emerging use cases in different IoT domains (e.g., smart transportation, smart industry). In this paper, we first report container migration use cases in the fog and discuss containerisation. We then provide a comprehensive overview of the state-of-the-art migration techniques for containers, i.e., cold, pre-copy, post-copy, and hybrid migrations. The main contribution of this work is the extensive performance evaluation of these techniques that we conducted over a real fog computing testbed. The obtained results shed light on container migration within fog computing environments by clarifying, in general, which migration technique might be the most appropriate under certain network and service conditions.

## 1. Introduction

The internet of things (IoT) [1] is a paradigm where each object, from a “smart” one (e.g., a smartphone, a wearable device) to a “dumb” one (e.g., a lamp post, a dumpster), can exchange data over the internet. Such objects may also store and process data, use sensors to perceive the surrounding environment, and modify the latter through actuators. In this scenario, people are active players in this ecosystem, as they consume and produce data through their smartphones and wearable devices. The number of smart objects connected to the internet exceeded the world human population in 2010 [2], and the McKinsey Global Institute estimates a potential economic impact for IoT applications of as much as $11.1 trillion per year in 2025 [3]. The great popularity that the IoT has been gaining is due to the fact that this paradigm is necessary for the implementation of many information and communications technology (ICT) services. However, IoT devices, and especially the wireless ones, typically have limited compute, storage, and networking resources and can be battery-powered [4]. As such, they are not sufficient in most cases to directly host resource-intensive services that perform complex computations over the big data [5] collected by sensors.

IoT devices often need to be integrated with more powerful resources, and fog computing [6] is emerging as a paradigm to provide them. Fog computing extends the cloud towards the network edge, distributing resources and services of computing, storage, and networking anywhere along the cloud-to-things continuum, close to where data are sensed and actions need to be performed. Depending on its requirements, a fog service may be deployed on: (i) resource-rich end devices, such as video surveillance cameras; (ii) advanced nodes at the network edge (e.g., gateways, cellular base stations); and (iii) specialised routers at the core network. Besides, fog services may be either stand-alone or can interact with one another and with cloud services to fulfil more complex use cases. As a result, IoT devices may perform only the lightest tasks and count on the fog for the most intensive ones [7]. In addition, the closer proximity of fog services to the end devices permits a set of advantages with respect to the exclusive dependence on the distant cloud, such as [8,9,10,11]: (i) low and predictable latencies; (ii) reduction in bandwidth consumption; (iii) better privacy and security; (iv) improved context awareness; and (v) uninterrupted services in the presence of intermittent or no network connectivity to the cloud. These benefits are indispensable to many emerging applications in several domains (e.g., smart healthcare, smart surveillance, smart transportation).

Virtualisation is an essential aspect of cloud as well as fog computing. Providing a cloud or fog service as a virtual environment (e.g., a virtual machine, a container) grants great elasticity and isolation, which in turn allows multi-tenancy and thus resource efficiency. Furthermore, migrating a service among nodes is another major feature that brings high flexibility and adaptability, as it is described in [12,13], where the authors mainly focus on virtual machine migration within cloud data centres. However, migration in the fog is influenced by some aspects that are not present in cloud-only environments. These aspects, which are as follows, occur when extending the cloud towards the network edge, indeed:

- Fog environments are characterised by a high **heterogeneity** of nodes in terms of hardware capabilities, architectures, and operating systems. Hence, there is the need for a virtualisation technology that is generic and lightweight enough to run on as many different types of fog nodes as possible;
- Fog nodes are interconnected through a wide area network (**WAN**) and therefore experience higher latencies and lower throughputs than those present within a cloud data centre. Based on this, it is beneficial during migration to transmit the lowest possible amount of data;
- In the cloud, the **total migration time** is only a secondary consideration. In the fog, instead, limiting it may be of paramount importance as there are situations in which protracted total migration times may lead to overall degraded performances [14];
- Most fog services, especially those deployed and running at the network edge, typically perform transient data analysis and time-critical control and are thus not supposed to write to any **persistent memory** (e.g., the disk), unlike cloud services. As a consequence, it is in general not necessary to transfer any persistent data during migration; what typically happens is that only the runtime (i.e., volatile) state is migrated and applied at destination to an available base service image representing the default disk state.

The focus of this paper is on **container migration in the fog**. This is a topical issue that promises to attract great attention in the near future for several reasons. Firstly, fog computing has been gaining much popularity as an enabler of emerging applications and services, especially in the IoT context. Secondly, containers are becoming a common virtualisation approach, within fog computing environments in particular, for their lightweight nature and overall performances [15,16,17]. Thirdly, container migration in the fog enables various use cases, both traditional ones (i.e., already present in cloud contexts) and emerging ones that are instead specific to fog computing, as we will further discuss. Nonetheless, container migration is still in its infancy, and hence only few works in literature provide knowledge in the field [17,18,19,20]. Besides, none of them evaluates and compares the existing container migration techniques. In our previous conference paper [21], we surveyed these techniques and attempted to point out their advantages and disadvantages, with specific attention to fog computing contexts. However, our claims were not supported by any experimentation. This article, instead, reports the extensive performance evaluation of the state-of-the-art container migration techniques that we carried out over a real fog computing testbed. The obtained results confirm most of the statements in [21], although we found some unexpected results and were able to analyse each aspect of container migration in depth. To the best of our knowledge, there is only one work in literature that makes a similar contribution [22]. Nonetheless, it only assesses a subset (i.e., cold and pre-copy migration) of the available techniques and concludes that container migration results in unexpected errors.

The rest of the paper is organised as follows. Section 2 describes the fog computing use cases where container migration may play an important role. Then, Section 3 points out the main virtualisation techniques, with specific attention to containerisation. In Section 4, we detail the characteristics of the existing container migration techniques. Next, Section 5 reports the experiments that we carried out along with the analysis of the obtained results. Finally, in Section 6, we draw the conclusions and outline the future work.

## 2. Motivation and Use Cases

Sensors and actuators can be connected to the internet either directly or through a sensor network that creates the necessary path. In any case, physical sensors and actuators exploit the concept of gateway, i.e., a device that on one side communicates with them and on the other side with the internet and the available services. Such a gateway is usually provided with computing and storage capabilities that allow code execution. Containers represent a valid solution for code execution (see Section 3), and their controlled migration is expected to play an important role in future fog-IoT deployments. Container migration will be mandatory in two different use cases: (i) mobility use case, i.e. to support use cases that natively require the service to migrate from one fog node to another for instance to support mobility of users and IoT devices [23]; (ii) management/orchestration use case, i.e. to enable dynamic management of resources and orchestration of services to ensure proper management of the fog computing infrastructure at large, in a similar manner cloud computing platforms operate [24].

**Mobility.** In specific use cases, migration will represent a mandatory function, required to properly support mobility of users or IoT devices [17]. In particular, migration will be crucial to handle scenarios in which sensors and actuators are natively characterised by mobility, such as when worn or brought by mobile users (e.g., wearables, smartphones) or carried by vehicles (e.g., car sensors). One scenario is the vehicular use case in which applications implementing real-time situation awareness or cooperative functionalities will require fog computing and storage capabilities to implement time-critical and data-intensive tasks [25]. For instance, let us consider an autonomous driving vehicle and a supporting service that collects context data from the environment (e.g., roadside sensors) and other vehicles to notify possible hazardous situations by applying mining techniques on the collected data. In this case, service migration is mandatory in order to keep the communication latency between the vehicle and the supporting service as low as possible and allow the vehicle to react promptly.

**Figure 1:**
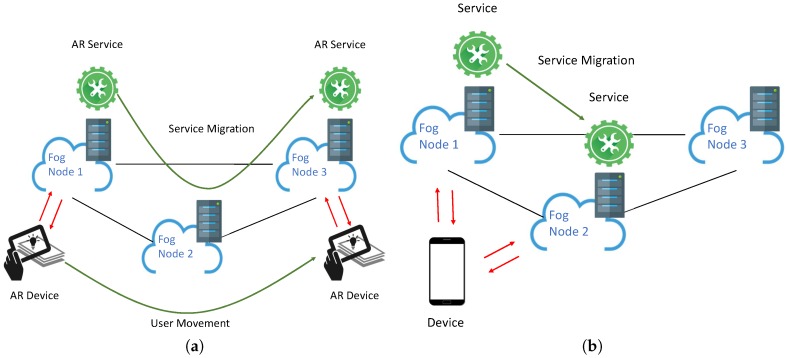
Container migration use cases: (**a**) mobility support; (**b**) platform management/orchestration.

Another scenario pertains applications using augmented reality (AR). In an industrial environment, portable AR devices (e.g., smart glasses, tablets) could be used by operators to obtain real time information on aspects such as: products, production procedures, instructions. Real-time AR systems exploit fog computing services to analyse and augment images with bounded low latency and without the need to offload them to the cloud [26]. In this case, for instance, migration could be exploited to support AR functionalities even when mobility is involved, as showed in Figure 1a. Let us consider an operator moving through the production plant for inspection equipped with smart glasses that show status information on the various equipment. In this case, the migration of the AR service is mandatory to ensure that images are always collected and analysed in due time.

**Management/orchestration.** The ultimate fog computing vision foresees an infrastructure in which single fog nodes or edge data centres (EDC) are seamlessly integrated with the cloud infrastructure. Such multi-layer infrastructure will require support for migrating services from the cloud to the fog and vice versa with the objective to accommodate application requirements that vary over time [27]. For instance, a service running in the cloud that requires low-latency interactions with cyber-physical systems for a certain period of time could be migrated from the cloud to a fog node, while a service running on a fog node that at a certain point demands for more resources can be offloaded to the cloud.

In addition to this feature, migration of services within the fog will represent an important feature to ensure the proper functionality of the fog infrastructure (see Figure 1b). Migration between different fog nodes or EDCs could be exploited to implement load balancing policies that aim at improving fog resource utilisation and at ensuring that application requirements are constantly met [28]. A management/orchestration service that controls load distribution on a fog infrastructure, for instance, could decide to migrate a service from one overloaded fog node to another in order to avoid service degradation. Conversely, the same management/orchestration service could decide to move out all the services from one fog node to others to enable its power saving mode. Such adaptive energy-aware computation offloading [29] could be implemented to mitigate the energy consumption of fog computing systems, which are expected to represent a significant share of the overall energy consumption of fog/cloud systems [30].

## 3. Virtualisation in the Fog

In this section, we give some background knowledge about the most popular virtualisation approaches, with specific attention to containerisation.

### 3.1. Virtualisation Approaches

Hardware virtualisation (also known as platform virtualisation) [31] is a set of techniques to support the creation of a so-called virtual machine (VM), which acts like a real computer able to run an operating system. More in general, software, when executed on VMs, does not have any access to, or visibility into, the underlying hardware resources of the host machine [32]. In platform virtualisation then, guest is another name for a VM, whereas the software (or firmware) in charge of VM instantiation on the host is called a hypervisor, or virtual machine monitor (VMM) [33]. Hardware-assisted virtualisation is a way for CPUs and other computer components to support virtualisation in hardware and thus improve virtualisation performance.

Operating system-level virtualisation [34], better known as containerisation, is a virtualisation approach enabled by a set of operating system features where the kernel itself allows the coexistence of multiple and isolated instances of user-space environments, leaving the hardware abstraction layer as well as the enforcement for process sandboxing to the shared kernel co-hosting them. Still, no limitations are in place in terms of choice for user-space, e.g., down to the C standard library if needed. Virtualisation instances of this kind, the so-called containers, still look like real computers from the point of view of programs running in them, as the latter can see select resources (file systems, networks, hardware devices and capabilities), i.e., those exposed by an ordinary operating system. However, software running inside a container can only see a subset of the resources made available by the kernel and specifically those assigned (intentionally) to the container.

**Figure 2:**
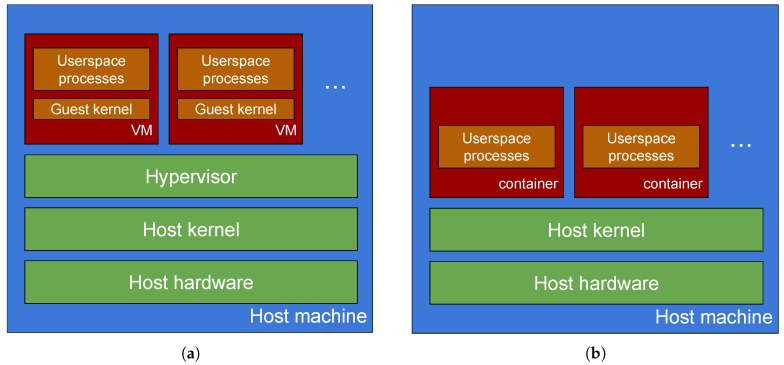
Hardware virtualisation (**a**) vs. containerisation (**b**).

The main difference between (platform) virtualisation and containerisation (see Figure 2) thus lies in the level at which abstraction and resource partitioning occurs, i.e., at the kernel level for the latter, below it for the former, and in the corresponding breadth of scope in reproducing the machine abstraction exposed to the guest environment, e.g., including emulation of low-level (firmware-based) hardware management interfaces for the former only. The requirements of virtualisation lead to higher-complexity implementations and higher storage requirements and are thus prone to substantial overhead, especially in case of unavailability of extensions for hardware-assisted virtualisation. This aspect, coupled with constraints of fog computing, where single-board computers (SBCs) or embedded systems are often involved whilst hardware-assisted virtualisation cannot always be taken for granted, makes VMs less suitable than containers for the fog computing domain in terms of overall footprint and support of workload mobility (i.e., migration) [15,16,17,35,36,37].

### 3.2. Containerisations

Containerisation takes many forms, according to the combination of techniques employed (and thus the level of control exerted on the guest environment) as well as the scope and usage [38]. Given that Linux is the most advanced example of an operating system featuring full support for containerisation, from now onwards the overview will refer to Linux-specific techniques, even if the same concepts have been applied elsewhere and the discussion does not otherwise lose generality. In terms of container-enabling techniques, the Linux kernel provides in particular control groups (typically abbreviated as cgroups), secure computing mode (typically abbreviated as seccomp), and namespacing. The latter implements kernel resource partitioning such that a set of processes is able to enumerate and access certain resources while another set of processes is given visibility on a different set of resources. This works by having the same namespace for the resources in the different process sets, while having those names actually point to distinct resources. Namespacing [39] is an approach first and foremost, and the Linux implementation provides interfaces to namespace a number of resources and subsystems: process/user/group IDs, filesystem resources, hostnames, routes, network interfaces, and interprocess communication descriptors, to name a few. Cgroups [40] is a kernel feature to limit, account for, and isolate usage of resources (e.g., CPU, memory, disk I/O, bandwidth) by a collection of processes. The seccomp [40] feature is a sandboxing facility first devised for grid computing users and originally intended as a technique to safely run untrusted compute-bound code. As such, it allows a process to make a one-way transition into a “secure” state from where system calls are disallowed, except for a very limited subset, restricted to (already) open file descriptors. In contrast to namespacing, seccomp does not virtualise (assigned) system resources but isolates the process from them entirely.

A middle ground between VMs and containers is represented by extremely lightweight and novel VMs that seamlessly plug into the containers ecosystem. An example of these are Kata Containers (see https://katacontainers.io/, accessed on 15 January 2019), which are managed by the OpenStack Foundation and merge technologies from Intel Clear Containers and Hyper runV. Specifically, their rationale is providing a user experience very similar to that of containers, while ensuring the portability and security advantages of VMs. This is achieved by featuring lightweight operating systems and kernels where isolation is enforced with the help of hardware-assisted (i.e., processor) extensions for hypervisors. Such extensions have been historically available on x86-architecture processors but are recently making their appearance on the ubiquitous ARM64 hardware.

As another example of performance enhancement technique, nvdimm (see https://github.com/qemu/qemu/blob/master/docs/nvdimm.txt, accessed on 15 January 2019) enables the guest to directly access (see https://git.kernel.org/pub/scm/linux/kernel/git/torvalds/linux.git/tree/Documentation/filesystems/dax.txt, accessed on 15 January 2019) the root file system from host memory pages, bypassing the guest page cache, by providing a memory-mapped persistent memory device to the VM hosting the container(s). Other so-called machine accelerators, at the moment x86-specific only, include skipping the firmware, e.g., basic input-output system (BIOS) or extensible firmware interface (EFI), in the guest when booting an ELF-format kernel (nofw) and reducing the interpretation burden for guest advanced configuration and power interface (ACPI) component (static-prt). These customisations together improve performance and boot time significantly. Finally, virtcontainers are highly optimised/accelerated VMs hosting (typically, but not solely) single-container workloads and an agent guest-side that enables interaction with the guest environment, e.g., command relaying for container life cycle management.

## 4. Container Migration Techniques

Container migrations may be either stateless or stateful. The former is so called because it causes the loss of the whole container state. Therefore, once on the destination node, the container restarts from scratch. As such, stateless migration simply consists in the following two steps: (i) the start of a new container on the destination node; and (ii) the deletion of the old container on the source node. **Stateful migration**, instead, is such that both the volatile and persistent states of the container are made available at destination once migration is completed. This paper focuses on stateful container migration. More specifically, in this section we provide an overview of the state-of-the-art techniques that may be adopted to statefully migrate containers, distinguishing between cold and live migration techniques. At the end of this section, Table 1 compares the discussed techniques in terms of what is transmitted in each migration phase.

### 4.1. Cold Migration

**Figure 3:**
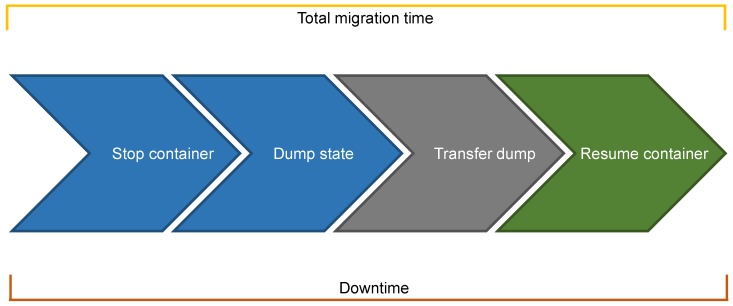
Cold migration.

The steps for **cold migration** are depicted in Figure 3, where the source node performs the blue steps, the destination node performs the green one, and the grey step involves both nodes. This migration technique is said to be “cold” because it: (i) first freezes/stops the container to ensure that it no longer modifies the state; (ii) then dumps the whole state and transfers it while the container is stopped; and (iii) finally resumes the container at destination only when all the state is available. As such, cold migration features a very long **downtime**, namely the time interval during which the container is not up and running. As shown in Figure 3, downtime even coincides with the **total migration time**, which is the overall time required to complete the migration. This aspect of cold migration goes against one of the main objectives of service migration in both the cloud and the fog, i.e., the limitation of the downtime. However, we highlight that this technique transfers each memory page only once, and this should significantly reduce both the total migration time and the overall amount of data transferred during migration.

Cold migration of containers, like all the other container migration techniques, is strongly based on checkpoint/restore in userspace (*CRIU*) (see https://criu.org/Main_Page, accessed on 14 January 2019). This started as a project of Virtuozzo (see https://virtuozzo.com/, accessed on 14 January 2019) for its OpenVZ containers but has been getting so much popularity over time that now it is used by all the most prominent container runtimes, such as runC. In detail, CRIU is a software tool for Linux that is written in C and mainly allows to: (i) freeze a running container; (ii) checkpoint/dump its state as a collection of files on disk; and (iii) use those files to restore the container and run it exactly as it was before being stopped. With respect to Figure 3, CRIU misses the third step, namely the state transfer to the destination node. Indeed, CRIU by itself only allows to restore a checkpointed container on the same host node; therefore, the actual state transfer has to be performed by exploiting tools such as *rsync* or secure copy protocol (*SCP*). Last but not least, we highlight that CRIU captures the runtime state of a container, namely all the memory pages and the execution state (e.g., CPU state, registers), but not the persistent one. However, this is in general not an issue within a fog computing environment, as we pointed out in the introduction of this paper, and might even represent a strength since less data are transmitted.

### 4.2. Live Migration

The main concern of live migration is the limitation of service downtime. Indeed, “live” means that the container keeps on running while most of its state is being transferred to the destination node. The container is typically suspended only for the transmission of a minimal amount of the overall state, after which the container runs at destination. When the downtime is not noticeable by the end user, live migration is said to be “seamless”. Three different live migration techniques exist for containers: pre-copy, post-copy, and hybrid.

#### 4.2.1. Pre-Copy Migration

**Figure 4:**
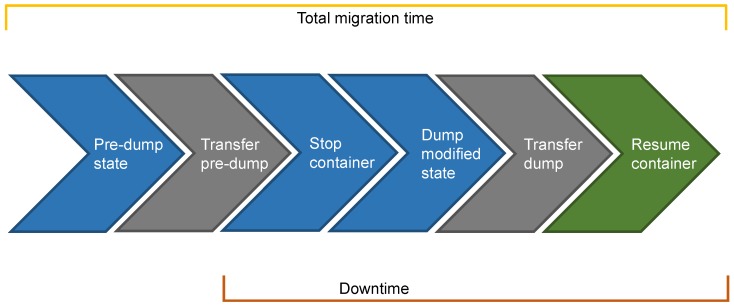
Pre-copy migration.

**Pre-copy migration** is so called because it transfers most of the state prior (i.e., *pre*) to freezing the container for a final dump and state transfer, after which the container runs on the destination node. It is also known as *iterative migration*, since it may perform the pre-copy phase through multiple iterations such that each iteration only dumps and retransmits those memory pages that were modified during the previous iteration (the first iteration dumps and transfers the whole container state as in cold migration). The modified memory pages are called **dirty pages**. Typically, iterations in the pre-copy phase are convergent, i.e., of shorter and shorter duration. If iterative, the pre-copy phase generally concludes when a predetermined number of iterations is reached. The container is then suspended on the source node in order to capture the last dirty pages along with the modifications in the execution state (e.g., changes in the CPU state, changes in the registers) and copy them at destination without the container modifying the state again. Finally, the container resumes on the destination node with its up-to-date state. Figure 4 shows the steps of pre-copy migration with a one-iteration pre-copy phase. The reason for this is that our current implementation of pre-copy migration, which is then evaluated in Section 5, presents only one pre-copy iteration. This implementation is based on CRIU, which provides all the basic mechanisms (e.g., the -*pre-dump* option) that are necessary to pre-dump the runtime state of a container and restore it afterwards (see https://criu.org/Iterative_migration, accessed on 15 January 2019). All the following considerations on pre-copy migration and hybrid migration (see Section 4.2.3) assume a one-iteration pre-copy phase.

The main difference between cold and pre-copy migrations lies in the nature of their dumps. The dump in cold migration represents the whole container state (as the pre-dump in pre-copy migration) and thus always includes all the memory pages and the execution state. The dump in pre-copy migration, instead, only includes those memory pages that were modified during the pre-copy phase, together with the changes in the execution state. As such, downtime for pre-copy migration should be in general shorter than that for cold migration because less data are transferred while the container is stopped. However, downtime for pre-copy migration is not deterministic, as it significantly depends on the number of dirty pages. Therefore, we expect pre-copy migration to be afflicted by the two factors that may increase the number of dirty pages: (i) the **page dirtying rate** featured by the container-hosted service, namely the speed at which the service modifies memory pages; (ii) the **amount of data** that are transferred during the pre-copy phase, since the more data are transferred in that phase, the more time the service has to modify pages. It is worth noting that both these factors should be always considered against the available **throughput** between the source and the destination node. Specifically, this is more obvious for the second factor, since this needs to be considered against the available throughput in order to estimate the time that the service has to modify pages during the pre-copy phase. With regard to the first factor, we expect that pre-copy migration performances are impaired by a page dirtying rate that starts approaching the available throughput (i.e., same order of magnitude), as in that case memory pages are modified at a rate which is comparable to that of page transfer. This would result in a state dump that is in general comparable to the state pre-dump or, in other words, to a downtime which is comparable to that for cold migration. As a final remark, we highlight that, unlike cold migration, pre-copy migration might transfer each memory page several times, with possible negative consequences on the overall amount of data transferred during migration and thus on the total migration time.

#### 4.2.2. Post-Copy Migration

**Figure 5:**
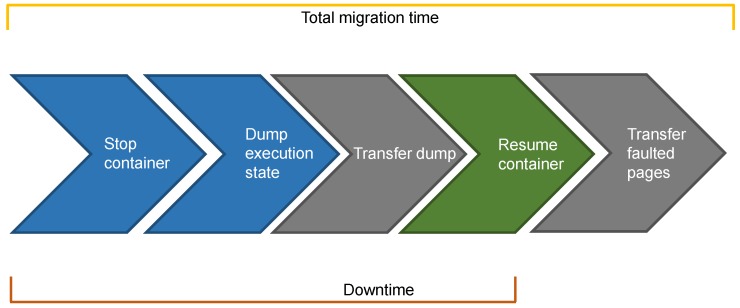
Post-copy migration.

**Post-copy migration** is the exact opposite of pre-copy migration. Indeed, it first suspends the container on the source node and copies the execution state to the destination so that the container can resume its execution there. Only after that (i.e., *post*), it copies all the remaining state, namely all the memory pages. Actually, there exist three variants of post-copy migration, which differ from one another on how they perform this second step. In this paper, we only describe the *post-copy migration with demand paging* variant, better known as **lazy migration** (see Figure 5), which is the only one that may be currently implemented using the functionalities provided by CRIU (see https://criu.org/Lazy_migration, accessed on 15 January 2019), e.g., the -*lazy-pages* and -*page-server* options. With lazy migration, the resumed container tries to access memory pages at destination, but, since it does not find them, it generates **page faults**. The outcome is that the *lazy pages daemon* at destination contacts the *page server* on the source node. This server then “lazily” (i.e., only upon request) forwards the faulted pages to the destination.

Post-copy migration copies each memory page only once. Therefore, it should transfer a data volume that is comparable with that of cold migration and with that of the pre-copy phase of pre-copy migration. Besides, similarly to cold migration, downtime for post-copy migration is irrespective of the page dirtying rate featured by the container-hosted service and of the overall amount of data that need to be transferred. This is due to the fact that the dump in post-copy migration is simply the execution state and does not contain any dirty memory pages. However, post-copy migration is afflicted by two drawbacks that are worthy of consideration. Firstly, **page faults degrade service performances**, as memory pages are not immediately available at destination once the container resumes. This could be unacceptable to the many latency-sensitive services present in fog computing environments. Secondly, during migration, this technique **distributes the overall up-to-date state** of the container between both the source and the destination node (before completion of post-copy migration, the source node retains all the memory pages, but some of them may be out-of-date because they have already been copied at destination and modified by the resumed container), whereas approaches like cold or pre-copy migrations retain the whole up-to-date state on the source node until the termination of the migration process. Therefore, if the destination node fails during migration, it may be no more possible to recover the up-to-date state of a post-copied container.

#### 4.2.3. Hybrid Migration

**Figure 6:**
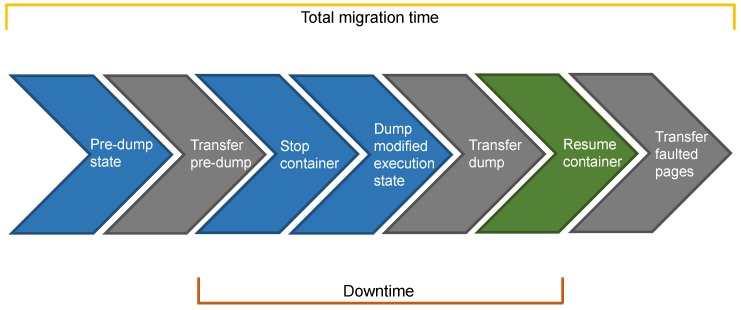
Hybrid migration.

As discussed in the previous sections, both pre-copy and post-copy migrations present some shortcomings: (i) pre-copy migration has a non-deterministic downtime; (ii) faulted pages in post-copy migration degrade service performances. **Hybrid migration**, which is illustrated in Figure 6, combines pre-copy and post-copy with the objective to subdue their limitations and sharpen their strengths. Going into detail, the first two steps of hybrid migration coincide with those of pre-copy migration, namely a pre-dump of the whole state and its transmission at destination while the container is still running on the source node. Then, the container is stopped, and its state is dumped in a way that combines the dumps of pre-copy and post-copy migrations. Indeed, the dump in hybrid migration is represented by the modifications in the execution state that occurred during the pre-copy phase. Once the dump is transferred to the destination node, the container can be restored. At this step, the destination node has the up-to-date container execution state along with all the memory pages. Nonetheless, some of them were dirtied during the pre-copy phase. As a result, the last step in hybrid migration consists in the page server at source lazily transmitting the dirty pages to the lazy pages daemon on the destination node. It is worth noting that the number of memory pages that are lazily transmitted in hybrid migration is generally less than that of post-copy migration since only the dirty pages are transferred. From now on, we will refer to these dirty pages as faulted pages, in line with the name used in post-copy migration to indicate the data transferred in this last phase. As pre-copy migration, we expect also hybrid migration to be affected by the page dirtying rate and the amount of data that are transmitted during the pre-copy phase. However, these factors should influence the total migration time for hybrid migration but not the downtime, as they alter the number of faulted pages. Moreover, hybrid migration is affected by the same two drawbacks of post-copy migration (see Section 4.2.2). To conclude, hybrid migration can be implemented by merging the CRIU options for pre-copy and post-copy migrations.

## 5. Performance Evaluation

In this section, we evaluate and compare the performances of the container migration techniques described in Section 4. The main objective is to determine whether there exists a technique that always performs the best or, otherwise, to delineate which technique might be the most suitable under certain network and service conditions. Section 5.1 illustrates the experiment setup, which consists in a real fog computing testbed. Next, Section 5.2 analyses the obtained results.

### 5.1. Experiment Setup

The overall testbed comprises two fog nodes and one end device. The former are two *Raspberries Pi 3 Model B* (i.e., ARMv8-A architecture) with Debian 9.5 and Linux kernel 4.14.73-v8+. They both run: (i) **CRIU 3.10** for the checkpointing and restore functionalities; (ii) **rsync 3.1.2** as file transfer mechanism; and (iii) **runC 1.0.1** as container runtime. Besides, they are both deployed within an office in the Department of Information Engineering of the University of Pisa. We chose Raspberries Pi as fog nodes because, even though they are able to host and run containers [36], they are rather limited in terms of hardware capabilities and thus represent the worst-case scenario in a fog environment. On the other hand, an *Asus ZenBook* notebook with Windows 10 emulates an IoT device that forwards data to a container-hosted fog service and receives actuation commands from it.

The core part of the experiment setup consisted in the appropriate tuning of the **throughput** between the fog nodes as well as in: (i) the choice of the **runtime state size** of the container (we highlight that, given the rsync settings described at the end of this section, the runtime state size represents the actual amount of data transferred during the pre-copy phase. Therefore, throughout Section 5, we use these two terms interchangeably); (ii) the selection of the values for the **page dirtying rate** of the container-hosted service. Indeed, as explained in Section 4.2.1 and Section 4.2.3, we expect pre-copy and hybrid migrations to be afflicted by these two factors, both to be considered against the available throughput. Instead, we do not expect these or any other specific factor to affect cold or post-copy migrations. By calibrating and combining the aforementioned factors, we implemented the following **four configurations**, which allow to carry out a comprehensive performance evaluation:

- **A**—this configuration presents a page dirtying rate and a throughput of different orders of magnitude, with the throughput higher than the page dirtying rate. However, given the throughput, the size of the runtime state leads to a prolonged pre-copy phase and thus gives the service plenty of time to modify memory pages. Therefore, this configuration is mainly aimed at evaluating the effects of a considerable runtime state size on the migration techniques;
- **B**—this configuration resembles configuration A in terms of runtime state size but features a page dirtying rate of the same order of magnitude of the available throughput. As such, this configuration is mainly aimed at investigating the effects of a high page dirtying rate on the migration techniques;
- **C**—this configuration shows a runtime state size that, considering the throughput, causes a shortened pre-copy phase and hence gives the service little time to modify memory pages. Besides, the page dirtying rate and the throughput are of different orders of magnitude, with the throughput higher than the page dirtying rate. Thus, the main objective of this configuration is to assess the migration techniques when both the factors are low, given the available throughput;
- **D**—this configuration resembles configuration C. The only difference is that the page dirtying rate in C and in D are of different orders of magnitude, with the former lower than the latter. However, the page dirtying rate in D is still lower than the throughput and of a different order of magnitude. The main purpose of this configuration is to estimate whether there are evident effects on the migration techniques when the page dirtying rate increases, though still being considerably lower than the throughput.

Going into detail, we achieved the previous four configurations by choosing and combining values as follows. Firstly, we selected two different throughput values that may both occur in real-life use cases within a fog environment (see Section 2). One throughput value may be present within a **mobility use case** where the destination fog node connects to the internet through 4G/LTE (e.g., a fog node within a public bus). The other throughput value, instead, may exist in a **management/orchestration use case** within the fog. Table 2 reports these values, together with the round trip time (RTT) values associated to them in the aforementioned use cases. Indeed, we also considered RTT values among the fog nodes in order to more accurately replicate the use cases network conditions in our testbed. To obtain the values presented in Table 2, we proceeded as follows. For the mobility use case, we considered a computer connected through Ethernet to the University of Pisa network as source fog node and a smartphone connected to the internet through 4G/LTE as destination. For the management/orchestration use case, instead, we employed two fixed computers belonging to a bridged LAN of the University of Pisa and installed in two different buildings placed about 1 km far apart. Throughput values were then obtained by performing 10 runs with the *iperf3* (See https://iperf.fr/, accessed on 30 December 2018.) tool, sending 50 MB each time. Similarly, RTT values were calculated over 10 runs, with 20 measurements per run. Since, in our testbed, the two Raspberries behaving as fog nodes are located in the same office, we had to emulate the values described in Table 2. Therefore, we exploited Linux Traffic Control Hierarchy Token Bucket (*tc-htb*) (see https://linux.die.net/man/8/tc-htb, accessed on 30 December 2018) to limit the throughput and Linux Traffic Control Network Emulator (*tc-netem*) (see https://www.systutorials.com/docs/linux/man/8-tc-netem/, accessed on 30 December 2018) to artificially set RTT values between the Raspberries.

We then chose the other values based on the identified throughput values. Specifically, we implemented a distributed application where both the client and the server are written in Java using the *Californium* (see https://www.eclipse.org/californium/, accessed on 15 January 2019) *CoAP* framework and thus required *openjdk8* to be installed on all the devices of the testbed. The server runs within a runC container in the fog and, once started, allocates 75 MB of RAM for random data. For our purpose, 75 MB is a suitable runtime state size; indeed, it determines a pre-copy phase that lasts tens of seconds with the lowest throughput and only few seconds with the highest one. The client, instead, runs on the Asus notebook and sends a POST request to the server every second to represent sensor data. Every time the server receives a request from the client, it modifies some of the memory pages with new random values. The server may perform this task with the following two page dirtying rates, which were identified by taking the throughputs into consideration. The lowest page dirtying rate is 10 KBps, which is about two orders of magnitude lower than the mobility use case throughput and about three orders of magnitude lower than the management/orchestration use case throughput. The highest page dirtying rate, instead, is 500 KBps, which is of the same order of magnitude of the throughput in the mobility use case and about one order of magnitude lower than the throughput in the management/orchestration use case. By combining the aforementioned values as reported in Table 3, we obtained the four configurations that were previously described. We highlight that all the implemented configurations share the same value of the runtime state size.

During the experiments, we evaluated all the migration techniques that are discussed in Section 4. In particular, we tested each technique five times for each of the four configurations. For each migration technique, we observed all the metrics that characterise it. As a result, the metrics that were overall observed are the following:

- **Pre-dump time**—taken in the pre-copy phase to dump the whole state on the source node while the service is still running;
- **Pre-dump transfer time**—needed in the pre-copy phase to transfer the generated pre-dump from the source to the destination node. It is not to be confused with the pre-dump time;
- **Dump time**—necessary in the dump phase to stop the container and dump its (modified) state on the source node. As described in Table 1, each migration technique presents a different concept of state dump;
- **Dump transfer time**—needed in the dump phase to transfer the generated dump from the source to the destination node. It is not to be confused with the dump time;
- **Resume time**—taken to restore the container at destination based on the state that was transferred up to that moment;
- **Faulted pages transfer time**—required in the last phase to transfer the faulted pages from the source to the destination node. Table 1 presents the different meanings that the term “faulted pages” assumes for post-copy and hybrid migrations;
- **Pre-dump size**—transferred during the pre-dump transfer time;
- **Dump size**—sent from the source to the destination node during the dump transfer time;
- **Faulted pages size**—transferred during the faulted pages transfer time.

We exploited the Linux *time* command to measure the times (i.e., the first six metrics) and the rsync -*stats* option to collect statistics regarding the amount of data transferred through rsync (i.e., the last three metrics). It is worth noting that we disabled the rsync data compression functionality during all the experiments. This was done because compressibility depends on data, and we did not want the experiment results to be influenced by this aspect. Similarly, we deleted the dump and the eventual pre-dump from the destination fog node after every experiment run. This was done to avoid that, by finding any of them at destination the next time, the incremental data transfer performed by rsync could transmit less data, thus influencing the experiment results. To conclude, we stored raw data in .csv files for the next phase of results analysis and plotting, which was performed in Python through *Plotly*, an open-source graphing library for Python.

### 5.2. Results

We now analyse and discuss the results obtained from the experiments. More specifically, Section 5.2.1 compares the migration techniques in terms of the total migration times; Section 5.2.2 evaluates the techniques with respect to the downtimes, while Section 5.2.3 does it with regard to the amounts of transferred data. As we will clarify in the following sections, each of these three metrics is the result of adding up some of the metrics from Section 5.1. To conclude, Section 5.2.4 summarises the main lessons learnt. All the following results are presented with a 95% confidence level.

#### 5.2.1. Total Migration Times

**Figure 7:**
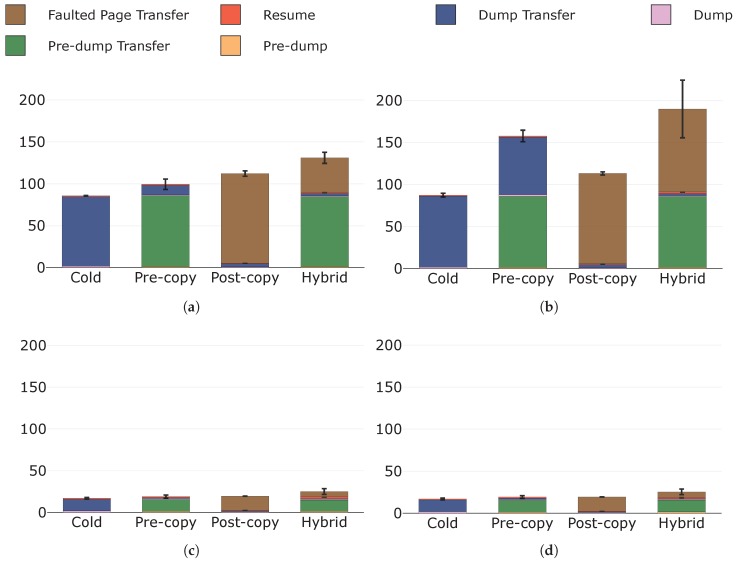
Total migration times (s) with their components in evidence under: (**a**) configuration A; (**b**) configuration B; (**c**) configuration C; and (**d**) configuration D.

Figure 7 depicts the total migration times, highlighting their components for each migration technique. It is evident how the times to perform local computations (i.e., pre-dump and dump the state, resume the container) are negligible with respect to those needed to transfer the state at destination (i.e., pre-dump transfer, dump transfer, and faulted pages transfer times). This is clearly more visible under configurations A and B, where the available throughput is significantly lower than that of configurations C and D.

Let us now compare the migration techniques. Cold migration presents the lowest total migration times, irrespective of the specific configuration. We were expecting this result, as cold migration transmits each memory page only once unlike pre-copy and hybrid techniques, which instead may transmit a memory page more than once (i.e., if it is dirtied). Less pages transferred result in a shorter total migration time, indeed. However, this is not always true. In Figure 7a, post-copy migration presents a longer total migration time than pre-copy, even though it transmits less data (see Section 5.2.3). Similarly, total migration times for post-copy migration are always longer than those for cold migration, even though these two techniques overall transmit similar amounts of data, as confirmed in Section 5.2.3. This unexpected result can be explained as follows. Post-copy migration is currently implemented according to the "lazy migration" variant, and hence faulted pages are transferred by the page server on the source node only upon request of the lazy pages daemon running at destination (see Section 4.2.2). Thus, **the time to perform such requests, which are not present in cold and pre-copy migrations, increases the overall total migration time**. This is particularly noticeable under configurations A and B, where RTT between the fog nodes is considerably higher than that of C and D.

Total migration times for cold and post-copy migrations are never influenced by an increase in the page dirtying rate. We expected this result as neither of these two techniques transfers dirty pages in any of its phases (see Table 1). Also pre-copy and hybrid migrations are not affected in terms of total migration time when page dirtying rate raises from configuration C to D. This important result shows how **there are no evident effects on the total migration times for pre-copy and hybrid migrations when the page dirtying rate increases, though still being lower and of a different order of magnitude from the throughput.** Besides, under these conditions, pre-copy migration performs similarly to post-copy in terms of total migration time. Nonetheless, the increment in the page dirtying rate from configuration A to B significantly prolongs the total migration times for pre-copy and hybrid migrations. Therefore, these two techniques are strongly affected in terms of total migration time by a page dirtying rate that, as for example under configuration B, reaches the same order of magnitude of the available throughput. The reason for this is that, under these conditions, the amount of pages dirtied during the pre-copy phase is comparable to that of pages transferred in that phase, namely to the whole state. This results in a state dump of considerable size for pre-copy migration and in a significant number of faulted pages for hybrid migration (see Table 1) and therefore in a substantial protraction of the total migration times.

Finally, we remark that hybrid migration always has the longest total migration times. This is because this technique inherits both the drawbacks of pre-copy and post-copy migrations in terms of total migration time, namely: (i) the fact that a memory page may be transferred more than once, as in pre-copy migration; (ii) the fact that also the time needed to request faulted pages needs to be considered, as in post-copy migration.

#### 5.2.2. Downtimes

**Figure 8:**
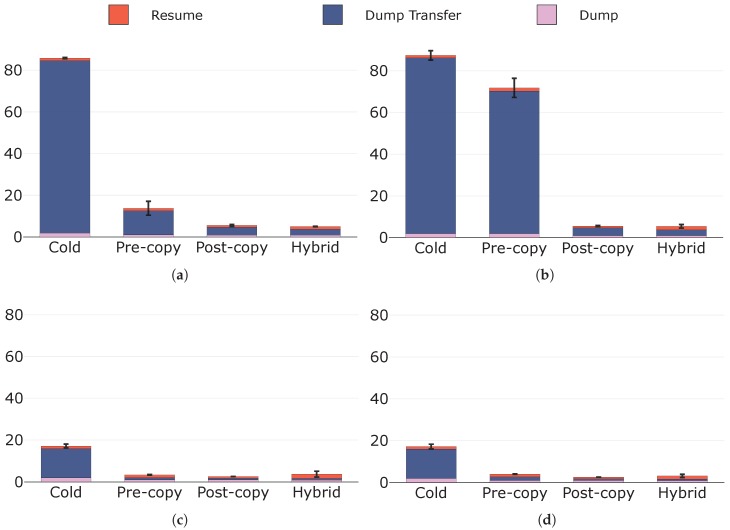
Downtimes (s) with their components in evidence under: (**a**) configuration A; (**b**) configuration B; (**c**) configuration C; and (**d**) configuration D.

Figure 8 depicts the downtimes for the migration techniques under the four considered configurations. As shown, the downtime for any technique is given by the sequence of the following times: (i) dump time; (ii) dump transfer time; and (iii) resume time. Cold migration always presents the highest downtime. This even coincides with the total migration time and proves to be unacceptable for most applications, especially for the critical ones that may exist in a Fog environment.

Under configurations C and D, the other three migration techniques show similar performances in terms of downtime. This is because, under these conditions, few memory pages are modified during the pre-copy phase; therefore, the dump size (and hence the dump transfer time) in pre-copy migration is comparable to those in post-copy and hybrid migrations. Besides, none of the four techniques seems to be affected in terms of downtime by an increase in the page dirtying rate from configuration C to D, as already noticed and commented with regard to the total migration times in Section 5.2.1. However, under configurations A and B, pre-copy presents significantly higher downtimes than post-copy and hybrid migrations. More specifically, under A, the downtime for pre-copy migration is longer because, considering the lower throughput, the size of the runtime state prolongs the pre-dump transfer time, giving the service more time to modify pages than under C or D. Therefore, the dump size in pre-copy migration grows as it strongly depends on the number of dirty pages, and the downtime does the same. A higher page dirtying rate under configuration B further increases the number of dirty pages and thus lengthens the pre-copy migration downtime. This even tends to that of cold migration, with the total migration time that, in addition, noticeably exceeds that of cold migration (see Section 5.2.1). It is evident, instead, how downtimes for post-copy and hybrid migrations are not influenced by the conditions characterising configurations A and B. This result was expected since neither of these two techniques includes dirty pages in its dumps, as reported in Table 1.

**Figure 9:**
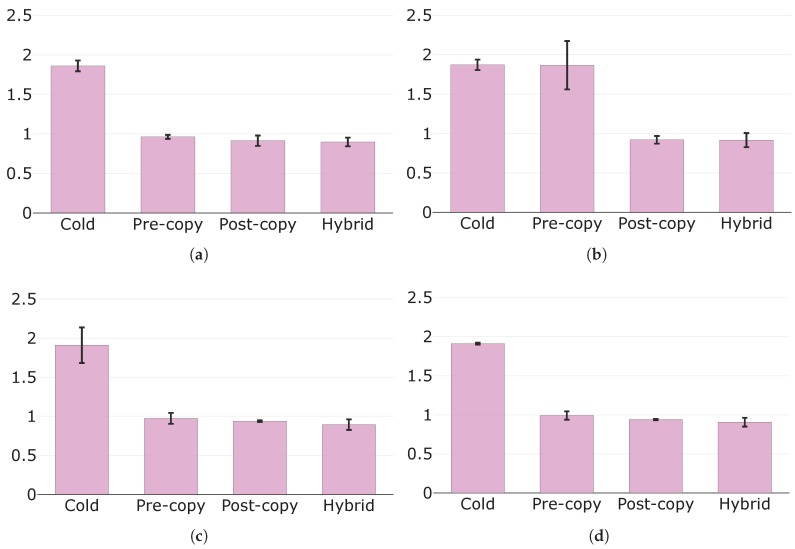
Dump times (s) under: (**a**) configuration A; (**b**) configuration B; (**c**) configuration C; and (**d**) configuration D.

**Figure 10:**
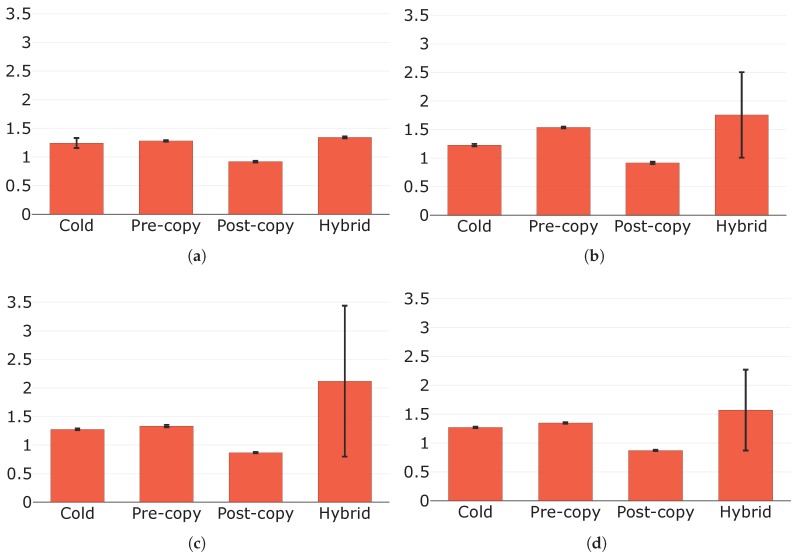
Resume times (s) under: (**a**) configuration A; (**b**) configuration B; (**c**) configuration C; and (**d**) configuration D.

We now analyse dump times and resume times in more depth. Both these times are not clear by looking at Figure 8; therefore, we illustrate them in Figure 9 and Figure 10, respectively. In general, dump times only depend on the amount of data that need to be dumped, while resume times depend on the amount of data from which the container must be restored. By looking at Figure 9, it is possible to notice how average dump times are always equal or less than 1 s except from those of cold migration (under all configurations) and that of pre-copy migration under configuration B. In these situations, indeed, the amount of data to be dumped (i.e., the dump size) is significantly higher than in all the others: the dump in cold migration is the whole container state, while that in pre-copy migration is of considerable size because of the conditions characterising configuration B. The condition on the runtime state size under configuration A, instead, is not sufficient on its own to cause an increase in the dump time for pre-copy migration. We also highlight that, with the only exception of pre-copy migration from configuration A to B, dump times are not affected by an increase in the page dirtying rate. This is due to the fact that, as reported in Table 1 and discussed in the previous sections, only the state dump in pre-copy migration includes dirty pages, and the increase in the page dirtying rate from configuration C to D does not determine an increase in the dump size that is significant enough to prolong the dump time.

Post-copy migration presents the shortest resume times (see Figure 10), as it restores the container at destination by only applying a very limited dump size to the base container image. All the other techniques have longer resume times. Going into detail, the cold and pre-copy techniques show similar values except from that of pre-copy migration under configuration B, which is caused by a higher amount of data to be applied to the base service image. We would have expected similar values also for hybrid migration; however, results show that, in general, resume times for this technique are greater. A possible explanation of this outcome is that jointly applying the pre-dump and the dump to the base image in hybrid migration is computationally more intensive.

#### 5.2.3. Transferred Data

**Figure 11:**
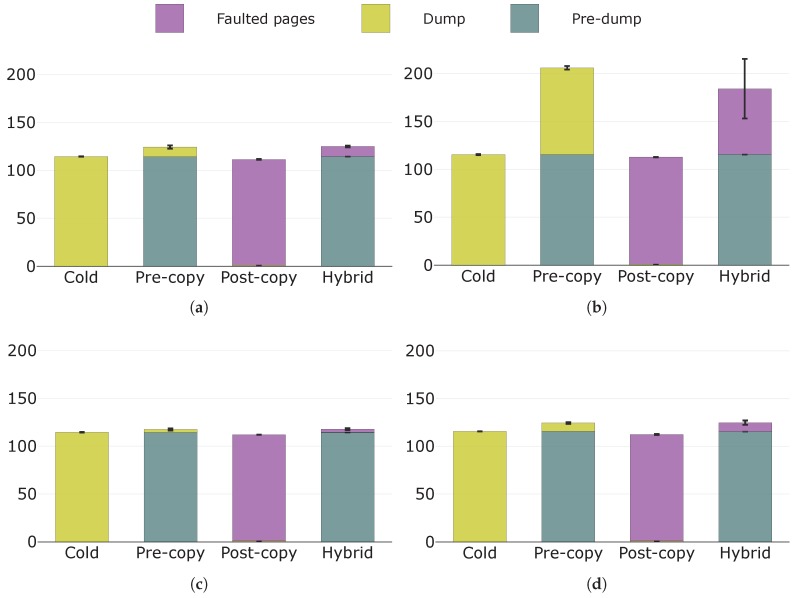
Amounts of transferred data (MB) with their components in evidence under: (**a**) configuration A; (**b**) configuration B; (**c**) configuration C; and (**d**) configuration D.

In Figure 11, we illustrate the amounts of data transferred during the experiments. Firstly, it is easy to notice how most of the transferred state is: (i) the pre-dump for pre-copy and hybrid migrations; (ii) the dump for cold migration; and (iii) the faulted pages for post-copy migration. This is in line with what reported in Table 1. Secondly, the thinness of the dump layer, which is almost invisible, in the bar charts of post-copy migration shows another detail: **the execution state of a container is markedly negligible with respect to the size of memory pages.** Another consideration that can be made by looking at Figure 11 is that **a container is an environment that occupies and updates more memory than that of the application running inside it**. Indeed, as discussed in Section 5.1, the server running in the container allocates 75 MB of RAM, but more than 110 MB are transferred on average during container migration. Similarly, the dump size for pre-copy migration and the faulted pages size for hybrid migration are greater than what we were expecting, given the two considered page dirtying rates.

Let us now compare the migration techniques. Cold and post-copy migrations transfer the lowest amounts of data, irrespective of the specific configuration. This is due to the fact that they both transfer each memory page only once. Under configurations A, C, and D, pre-copy and hybrid migrations generate volumes of data that are comparable to those of cold and post-copy migrations, even though slightly higher. Going into detail, pre-copy and hybrid migrations perform at their best under configuration C, where there is the maximum difference between the throughput and the page dirtying rate (i.e., about three orders of magnitude) and, considering the available throughput, the runtime state size leads to a limited pre-copy phase. An increase in the page dirtying rate from configuration C to D augments the amounts of transferred data only for pre-copy and hybrid migrations, but these increases are limited because the page dirtying rate is still of a different order of magnitude from the throughput. Instead, under B, a page dirtying rate of the same order of magnitude of the throughput causes these two migration techniques to modify a quantity of memory pages that is comparable to the whole state and thus transfer significantly greater volumes of data than those of cold and post-copy migrations. In particular, the dump size is what grows in pre-copy migration and the faulted pages size is what increases in hybrid migration, as these are the parts of the state containing dirty pages (see Table 1).

#### 5.2.4. Lessons Learnt

For convenience, in Table 4 we summarise the most salient results relative to the total migration time, the downtime, and the amount of transferred data. As a closing remark, this work shows that **no migration technique is the very best** under all network and service conditions. However, based on the discussed results, we can conclude by stating that in general:

- **Cold** migration is to be avoided under all conditions because it always causes downtimes that are considerably higher than those of the other techniques;
- In situations where the throughput between nodes and the page dirtying rate are of different orders of magnitude, with the former higher than the latter, and the pre-copy phase does not have a prolonged duration (e.g., under configurations C and D), **pre-copy** migration may be the best option. Indeed, it has similar performances to those of post-copy and hybrid migrations, but it is not afflicted by the issues characterising these other two techniques (see Section 4.2.2);
- In situations where the page dirtying rate is of the same order of magnitude of the throughput and/or the pre-copy phase has a prolonged duration (e.g., under configurations A and B), pre-copy is to be avoided mainly because of rather long downtimes. **Post-copy** could be the best alternative, considering that it provides downtimes comparable to those of hybrid migration but performs better in terms of total migration time and amount of transferred data. It is worth noting, though, that post-copy presents a couple of non-negligible issues, which are explained in Section 4.2.2.

## 6. Conclusions

Containerisation and container migration are fundamental aspects of fog computing, a paradigm that provides resources and services of computing, storage, and networking near to where sensors and actuators are deployed. In this paper, we critically analysed the existing container migration techniques, with a specific focus on their suitability for fog computing environments. We carried out a comprehensive performance evaluation of these techniques over a real fog computing testbed, observing their behaviour under different conditions of throughput and page dirtying rate. After the assessment of total migration time, downtime, and overall amount of transferred data, we can conclude that no container migration technique is the very best under all network and service conditions. Therefore, we identified categories of conditions and pointed out which technique could be the most suitable under each of those categories. The results show that, in general, cold migration is mostly afflicted by long downtimes, while the main shortcoming of hybrid migration is a prolonged total migration time. Pre-copy and post-copy migrations might therefore represent the best options, under different conditions.

As a future work, we plan to evaluate and compare container migration techniques also within cloud computing data centres, where conditions are different than those in fog environments (e.g., throughputs are in general much higher, there is usually the need to also migrate the persistent state). In addition, we would like to propose a novel migration approach that better adapts to the diverse network and service conditions.

## Figures and Tables

**Table 1:** What is transferred in each phase of the migration techniques.

Technique	Pre-Dump	Dump	Faulted Pages
Cold		Memory pages and execution state	
Pre-copy	Memory pages and execution state	Dirty pages and changes in execution state	
Post-copy		Execution state	Memory pages
Hybrid	Memory pages and execution state	Changes in execution state	Dirty pages

**Table 2:** Considered throughput and round trip time (RTT) values (95% confidence intervals).

Use Case	Throughput (Mbps)	RTT (ms)
Mobility	11.34±2.31	122.95±5.57
Management/Orchestration	72.41±3.87	6.94±0.61

**Table 3:** How values were combined to obtain the four configurations.

Throughput and RTT	Page Dirtying Rate	Configuration
Mobility	Low	A
Mobility	High	B
Management/Orchestration	Low	C
Management/Orchestration	High	D

**Table 4:** Summary of the evaluated migration techniques.

Technique	Total Migration Time	Downtime	Transferred Data
Cold	Always the lowest.	Always the highest. Coincides with the total migration time.	Always the least. Comparable to those of post-copy.
Pre-copy	Comparable or lower than that of post- copy when page dirtying rate and throughput are of different orders of magnitude, with page dirtying rate lower than throughput. Higher than that of post-copy when page dirtying rate is of the same order of magnitude of throughput.	Higher than those of post-copy and hybrid when page dirtying rate is of the same order of magnitude of throughput and/or pre-copy phase has a prolonged duration.	Much more than cold or post-copy when page dirtying rate is of the same order of magnitude of throughput.
Post-copy	Higher than that of cold, especially with a very high RTT between nodes. Comparable or higher than that of pre- copy when page dirtying rate and throughput are of different orders of magnitude, with page dirtying rate lower than throughput. Lower than that of pre-copy when page dirtying rate is of the same order of magnitude of throughput.	Always low and comparable to that of hybrid.	Always the least. Comparable to those of cold.
Hybrid	Always the highest.	Always low and comparable to that of post-copy.	Much more than cold or post-copy when page dirtying rate is of the same order of magnitude of throughput.
